# Structural alterations of brain in different disease states of Crohn's disease: Results of a cross-sectional study in a Chinese hospital

**DOI:** 10.1016/j.heliyon.2024.e27446

**Published:** 2024-03-07

**Authors:** Mengting Huang, Hui Ma, Yan Zou, Wenliang Fan, Lei Tu, Jie Zhao, Guina Ma, Nan Diao, Xin Li, Ping Han, Liangru Zhu, Heshui Shi

**Affiliations:** a Department of Radiology, Union Hospital, Tongji Medical College, Huazhong University of Science and Technology, Wuhan, 430022, China; b Hubei Province Key Laboratory of Molecular Imaging, Wuhan, 430022, China; c Division of Gastroenterology, Union Hospital, Tongji Medical College, Huazhong University of Science and Technology, Wuhan, China

**Keywords:** Voxel-based morphometry, Gray matter volume, Neuroimaging, Crohn's disease

## Abstract

**Rationale and objectives:**

To investigate alterations in the brain structure in patients with Crohn's disease in activity (CD-A) and in remission (CD-R) compared to healthy controls (HCs) and explore the relationship between gray matter volume (GMV) and psychological disorders.

**Materials and methods:**

A total of 127 CD patients (62 CD-A, 65 CD-R) and 92 healthy controls (HCs) were enrolled and analyzed in this study. The Crohn's disease activity index (CDAI) was used as the grouping criteria. Voxel-based morphometry (VBM) was applied to investigate gray matter volume (GMV), white matter volume (WMV) and global cerebrospinal fluid (CSF) volume alterations. Pearson correlation analysis was used to evaluate the relationships.

**Results:**

The CSF volume was negatively correlated with the disease duration in CD-R. Increased GMV of CD was observed in the parahippocampal gyrus, precentral gyrus, precuneous cortex, and subcallosal cortex, decreased was located in the occipital pole, precentral gyrus, inferior temporal gyrus, middle frontal gyrus, angular gyrus, frontal pole, lateral occipital cortex, and lingual gyrus. The GMV in the right temporal pole, left precuneous cortex, and left cingulate gyrus had a positive correlation with erythrocyte and hemoglobin in CD groups. The GMV in the right frontal pole, right postcentral gyrus, and left cingulate gyrus had a negative correlation with somatization in the CD groups. The GMV in the right temporal pole had a negative correlation with psychoticism and other in the CD groups. The GMV in the left cingulate gyrus was positive with bowel symptoms and systemic symptoms in the CD groups.

**Conclusion:**

Alterations of GMV in CD-A and CD-R and associated correlation with psychological disorders may provide evidence for possible neuro-mechanisms of CD with psychological disorders.

## Highlights

• The sample size is the largest in the literature on the brain structure in CD.
• VBM gray matter volume differences were significant for the disease activity.
• Gray matter volume correlated with bowel and systemic symptoms.
• Significant correlations were found between psychological disorders scales and GMV.
• Significant correlations were found between SCF volume and disease duration.

## 1 Introduction

Crohn's disease (CD) is a chronic and recurrent gastrointestinal inflammatory disease [1,2], and the incidence has increased significantly in recent years [3]. The clinical manifestations of CD are diverse, including gastrointestinal manifestations and extraintestinal manifestations, such as abdominal pain, diarrhea, perianal fistula, arthralgia, and fatigue [[4], [5], [6]]. Meanwhile, chronic and recurrent inflammatory disease has posed an economic burden to the patients of CD in the quality of life. Some evidence suggests that CD is associated with neuropsychiatric symptoms, such as poor emotional functioning, anxiety, and depression [7,8]. Nevertheless, the causes of these clinical and neuropsychiatric symptoms have not been fully demonstrated, especially their effects on the central nervous system (CNS). Intestinal inflammation and neuropsychiatric symptoms may activate sensitization pathways in the central nervous system that transmit visceral injurious afferent signals from the gut to the brain [9], affecting symptom perception and intestinal function [10]. Some studies have demonstrated that the structure and neurochemical composition of the intestinal nervous system in active inflammatory bowel disease have altered [11]. The brain-gut axis may provide a feasible hypothesis for the relationship between intestinal symptoms and the CNS in patients with CD [12].

Structural magnetic resonance imaging (MRI) provides relatively fine brain microstructure information and has been widely used to capture the spatial patterns of key brain regions in patients with various diseases [13,14]. To date, morphometry of MRI has been used to evaluate brain structural alterations among patients of CD in remission. The alterations of gray matter volume (GMV) in CD patients were tested by voxel-based morphometry (VBM) and cortical thickness analysis. VBM is a topological structure analysis technique, which has been used in disorders such as schizophrenia, Parkinson's disease, and ulcerative colitis [15]. For instance, Agostini et al. has been reported that the GMV of CD patients in the dorsolateral prefrontal cortex and anterior midcingulate cortex were altered compared to healthy controls (HCs), which were negatively correlated with the disease duration [16]. There are several studies of the relationship between clinical symptoms and psychological disorders such as fatigue, abdominal pain, anxiety, and depression with brain morphometry in CD. Bao et al. reported that the GMV was found to be decreased in the anterior cingulate cortex (ACC) of abdominal pain in CD patients compared to those without abdominal pain and HCs [17]. In addition, another study found that a decrease in GMV and cortical thickness of the anterior cingulate cortex in CD patients was observed along with an increase in local gyrification index [18]. In our previous research, we found that the regional homogeneity of CD patients in frontal, putamen, postcentral, supplementary motor area, and temporal regions was altered, and were associated with psychological disorders symptoms [19]. In conclusion, these results suggest that abnormal processing of the brain's nervous system may be the related pathophysiological mechanism of CD.

However, studies of GMV structural changes in CD remain limited, particularly in patients with CD in activity (CD-A) and patients with CD in remission (CD-R) compared to HCs. Therefore, in this study, we expanded the study sample size. We aim to (a) explore possible group differences in GMV between the CD-A, CD-R, and HCs by the VBM method; and [2] assess relationships between the structure alterations clinical data and psychological data.

## 2 Material and methods

### 2.1 Ethics statement

The present study was approved by the institutional ethics committee of Tongji Medical College of Huazhong University of Science and Technology, and conducted by the Union Hospital, Tongji Medical College, Huazhong University of Science and Technology. All participants were informed of the experimental procedure and signed informed consent forms.

### 2.2 Subjects

**Fig. 1:**
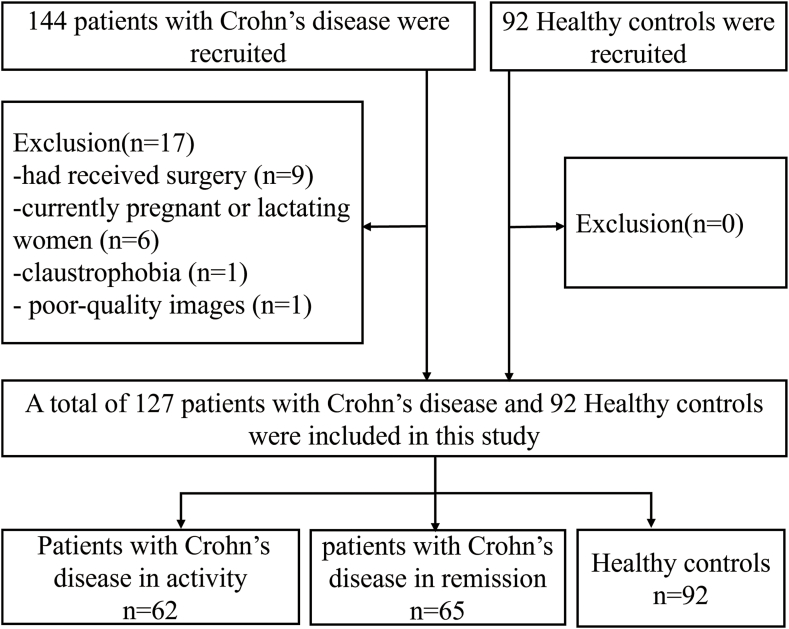
Flow diagram of the enrolled patients. Note: CD-A refers to patients with Crohn's disease in activity. CD-R refers to patients with Crohn's disease in remission. HCs refer to Healthy controls.

One hundred and twenty-seven patients with CD and ninety-two HCs were consecutively enrolled in this study. Patients with CD met the inclusion criteria as follows [1]: being over 18 years old [2]; right-handed [3]; native Chinese speaker; and [4] education level of more than 9 years. The criteria for the disease activity were the Crohn's disease activity index (CDAI) scores [20]. CD-R refers to CD patients in remission, for which the CDAI score is less than 150. Conversely, CD-A refers to CD patients in activity, in which the CDAI score is ≥150. At the same time, these patients also underwent imaging assessments (computer tomography enterography or magnetic resonance imaging enterography), such as wall thickness, locations of the lesion, comb sign and enlarged lymph nodes. HCs met the inclusion criteria as follows [1]: matching with patients by gender and age as much as possible [2]; no disease manifestations of the digestive system [3]; the same with points [[2], [3], [4]] on CD. The exclusion criteria of patients and HCs were as follows [1]: receiving CD-related abdominal surgery or active corticosteroid use [2]; with claustrophobia or metal implants [3]; using psychotropic drugs or opioids in the past 3 months [4]; suffering head trauma, tumor or loss of consciousness [5]; pregnant or lactating women [6]; having chronic diseases such as hypertension and diabetes that may affect the structure of the brain; and [7] having poor quality images. The flow diagram of the enrolled patients with CD and HCs is shown in Fig. 1. We collected the clinical and psychological features of a clinical database and psychological questionnaire. The questionnaire includes the Inflammatory Bowel Disease Questionnaire (IBDQ), Symptom Checklist-90 (SCL-90), and Social Support Rating Scale (SSRS) (Supplementary Material 2–4). Additionally, these questionnaires were used for the HCs who were included in this study.

### 2.3 MRI data acquisition

All MRI data were acquired using a 3.0T SIEMENS scanner (Skyra, Germany) equipped with a 32-channel head coil. The T1-weight images were acquired for magnetization prepared rapid gradient echo (MP2RAGE) sequence, parallel acquisition technique GRAPPA acceleration factor 3. The scanner parameters were as follows: voxel size was 1 × 1 × 1 mm; time of repetition (TR) was 5000 ms; time of echo (TE) was 2.98 ms; field of view (FOV) was 256 mm × 240 mm; flip angle (FA) was 9°; bandwidth was 240 Hz/Px; slice thickness was 1.0 mm (no gaps); and slices was 176. The total scan time was 8′22″.

### 2.4 MRI data preprocessing

The structural MRI data were pre-processed in the DPARSF packages of DPABI software (http://rfmri.org/dpabi), which was implemented in MATLAB 2017a (Mathworks).

The preprocessing steps were as follows, which were pre-processed in the CAT12 of SPM software (https://www.fil.ion.ucl.ac.uk/spm) [1]: segmentation (the brain images were segmented into gray matter (GM), white matter (WM), and cerebrospinal fluid (CSF)) [2]; normalization (the brain images were converted into Montreal Neurological Institute (MNI) standard space and re-sampled to 1.5 × 1.5 × 1.5 mm^3^) [3]; modulate (the images were modulated in MNI space) [4]; smooth (the resultant GM images were smoothed through a Gaussian kernel of 8 mm full-width at the half maximum). Meanwhile, we also did a quality check on the brain images before further analysis, which excluded poor-quality images. In the preprocessing, we calculated the gray matter volume (GMV), total intracranial volume (TIV), and CSF volume of the whole brain. The statistical module was in DPABI.

### 2.5 Statistical analysis

The clinical data and demographics were computed by IBM SPSS Statistics 26.0 software and GraphPad Prism 8 software. One-way analysis of variance (ANOVA), followed by a post hoc two-sample *t*-test was used for comparison. The statistical module of DPABI was utilized to assess voxel with a significant level of voxel P < 0.001 and cluster P < 0.01 (GRF corrected). Gender, age, and TIV were the covariates. The One-way ANOVA and two independent-sample T-tests were applied to evaluate the normally distributed continuous variables of the clinical and demographics data of patients with CD and HC. The masks of differential brain regions were created from the results of the One-way ANOVA, followed by a post hoc two-sample *t*-test using the extraction tool (DPABI, Utilities, ROI Signal Extractor), and the GMV values of brain regions were extracted. Pearson's correlation was used to evaluate the relationship between the GMV values that were extracted, clinical data, and psychological assessment scores. P < 0.05 was considered statistically significant, and P values were two-tailed.

## 3 Results

### 3.1 Demographics and clinical characteristics

Results of the clinical and demographic characteristics in patients with CD and HCs group are summarized in Table 1. There are no significant differences in age, gender, BMI, education disease duration and medication, marital status and blood mineral among the participants. Compared to the CD-R, the CD-A has a higher value of C-reactive protein (CRP) (P = 0.001), erythrocyte sedimentation rate (ESR) (P = 0.001), platelet levels (P = 0.011) and erythrocyte (P = 0.037). The bowel symptoms scores (P = 0.0003), emotional function scores (P = 0.0005) and social impairment scores (P = 0.006) of IBDQ, somatization (P = 0.010), interpersonal sensitivity scores (P = 0.001), depression scores (P = 0.0007), and bigoted scores (P = 0.0005) of SCL-90 of CD-A group were significantly different with CD-R group (Supplementary Material Table 1).

**Table 1:** Clinical and demographics data of patients with Crohn's disease and healthy controls.

Characteristics	CD-A (n = 62)	CD-R (n = 65)	HCs (n = 92)	P
Sex (Male/Female)	50/12	49/16	66/26	0.454
Age (year)	31.12 ± 12.34	30.55 ± 11.14	28.65 ± 9.49	0.328^a^
Body Mass Index	19.77 ± 3.79	20.00 ± 4.04	19.98 ± 3.37	0.925^a^
Education	16.72 ± 2.31	16.75 ± 3.61	17.40 ± 2.53	0.233^a^
Disease Duration (years)	1.21 ± 1.13	1.50 ± 2.47	–	0.400^b^
C-reactive protein	24.38 ± 30.22	10.26 ± 15.91	–	0.001^b^
Erythrocyte sedimentation rate	21.87 ± 19.35	11.00 ± 13.22	–	0.001^b^
platelet levels	299.45 ± 108.62	251.25 ± 103.71	–	0.011^b^
Crohn's Disease Activity Index	273.66 ± 35.92	81.24 ± 23.96	–	0.001^b^
erythrocyte	4.49 ± 0.55	5.60 ± 1.93	–	0.001^b^
hemoglobin	120.03 ± 25.37	123.63 ± 21.19	–	0.386^b^
hematokrit	38.48 ± 4.63	37.58 ± 5.74	–	0.334^b^
Osmolality	297.28 ± 3.24	298.42 ± 3.27	–	0.051^b^
Na+	140.42 ± 1.50	140.59 ± 1.37	–	0.505^b^
K+	4.08 ± 0.30	4.10 ± 0.31	–	0.712^b^
Montreal classification	–	–	–	–
L1:L2:L3:L4	16:7:34:5	15:7:39:4	–	0.937^c^
B1:B2:B3:P	15:20:27:31	24:15:26:23	–	0.277^c^
Current therapy	–	–	–	0.001^c^
Nutritional therapy	0	5		
5-aminosalicylates	12	27	–	
biological therapy	24	21	–	
Combined therapy	26	12	–	
Marital status	–	–	–	0.567^c^
Married/cohabitating	35	32	48	
Widowed/divorced	5	4	4	
Single	22	29	40	

Note: CD-A refers to patients with Crohn's disease in activity. CD-R refers to patients with Crohn's disease in remission. HCs refer to Healthy controls. L1, ileum; L2, colonic; L3, ileocolonic; L4, upper gastrointestinal tract disease; B1, on inflammatory and no penetrating; B2, inflammatory; B3, penetrating; P, perianal disease.

a P value for One-way ANOVA.

b P value for a Two-sample *t*-test.

c P value for χ2 test.

### 3.2 Altered whole-brain structural morphometry in CD-A and CD-R compared to HCs

**Fig. 2:**
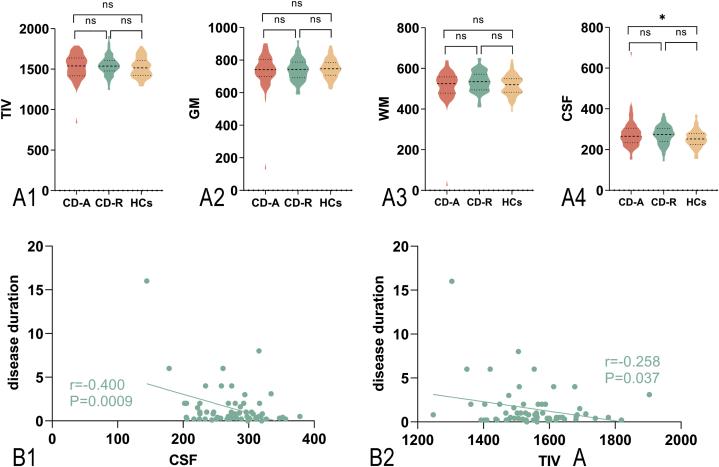
Altered whole-brain structural morphometry in patients with Crohn's disease compared to HCs. Note: CD-A refers to patients with Crohn's disease in activity. CD-R refers to patients with Crohn's disease in remission. HCs refer to Healthy controls. TIV = total intracranial volume; GM = gray matter; CSF = cerebrospinal fluid. (A) Altered whole-brain structural morphometry in CD-A and CD-R compared with HCs. TIV: CD-A = 1529.37 ± 158.01, CD-R = 1545.26 ± 117.69, HCs = 1518.73 ± 117.47, P = 0.455; GM:CD-A = 736.39 ± 107.10, CD-R = 738.77 ± 66.56, HCs = 748.30 ± 57.12, P = 0.589; WM: CD-A = 514.96 ± 82.03, CD-R = 535.11 ± 50.41, HCs = 517.74 ± 48.62, P = 0.115; CSF: CD-A = 278.02 ± 74.47, CD-R = 271.37 ± 45.99, HCs = 252.68 ± 41.33, P = 0.038. (B) Correlation of duration with TIV and CSF volume in patients of Crohn's disease in remission.

There is no significant difference in TIV (CD-A, 1529.37 ± 158.01; CD-R, 1545.26 ± 117.69; HCs, 1518.73 ± 117.47; P = 0.455), GM volume (CD-A, 736.39 ± 107.10; CD-R, 738.77 ± 66.56; HCs, 748.30 ± 57.12; P = 0.589), and WM volume (CD-A, 514.96 ± 82.03; CD-R, 535.11 ± 50.41; HCs, 517.74 ± 48.62; P = 0.115). A significant increase in CSF volume was evident in CD-A and CD-R compared to HCs (CD-A, 278.02 ± 74.47; CD-R, 271.37 ± 45.99; HCs, 252.68 ± 41.33; P = 0.038) (Fig. 2A). We also discovered that the disease duration in CD-R was negatively correlated with the CSF volume (r = −0.400, P = 0.0009) and the TIV (r = −0.258 P = 0.037) (Fig. 2B).

### 3.3 Altered regional structural morphometry in CD-A and CD-R compared with HCs

**Fig. 3:**
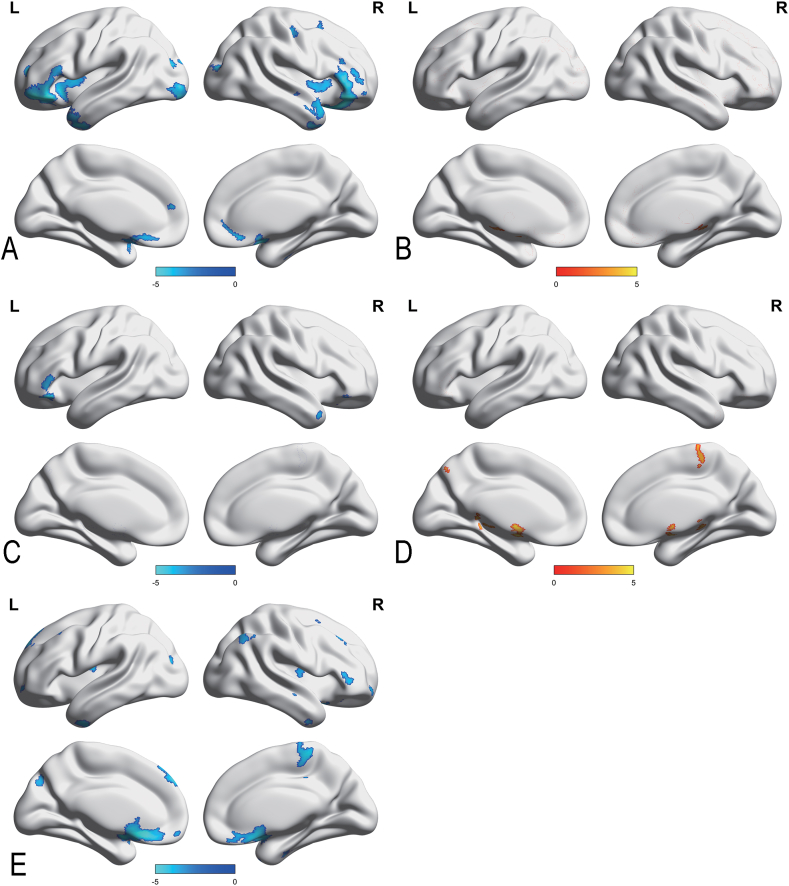
Brain regions with significant differences in gray matter volume between patients of Crohn's disease and healthy controls. Note: The results employed gender, age, and intracranial volume as covariates. Significant increased (red) and decreased (blue) (GRF corrected, voxel p < 0.001, cluster p < 0.01). The color bar represents the t-value. (A) Brain regions with significant reduction in gray matter volume (GMV) between patients with Crohn's disease in activity (CD-A) and healthy controls (HCs). (B) Brain regions with significant increase in GMV between CD-A and HCs. (C) Brain regions with significant reduction in GMV between patients with Crohn's disease in remission (CD-R) and HCs. (D) Brain regions with significant increase in GMV between CD-R and HCs. (E) Brain regions with significant reduction in GMV between CD-A and CD-R.

As shown in Table 2, a significant reduction in GMV was evident in the left occipital pole, right precentral gyrus, left inferior temporal gyrus, right middle frontal gyrus, left angular gyrus, left frontal pole, lateral occipital cortex, left lingual gyrus in CD-A compared to HCs with gender, age, and TIV as covariates. Conversely, CD-A had significantly greater GMV in parahippocampal gyrus compared to HCs (Fig. 3A–B). Meanwhile, the CD-R showed significantly decreased GMV in the left frontal orbital cortex, right frontal pole, and right temporal pole with gender, age, and TIV as covariates. The GMV of CD-R in the left precuneous cortex, right precentral gyrus, and left subcallosal cortex was increased than HCs (Fig. 3C–D). Compared with CD-A, CD-R exhibited decreased GMV of the frontal pole, right postcentral gyrus, inferior temporal gyrus, lateral occipital cortex, left central opercular cortex, left cingulate gyrus, left middle frontal gyrus, left superior parietal lobule, left precuneous cortex, left lingual gyrus, and supramarginal gyrus (Fig. 3E).

**Table 2:** Brain regions with significant differences in gray matter volume between patients with Crohn's disease and healthy controls.

Regions	BA	Hem	MNI peak coordinate			Voxel size	T value
			X	Y	Z		
CD-A < HCs							
Occipital pole	18	L	−33	−93	−12	1759	−4.804
Precentral gyrus	6	R	42	−11	50	692	−3.077
Inferior temporal gyrus	20	L	−48	−47	−20	256	−3.501
Middle frontal gyrus	44	R	47	12	35	167	−3.537
Angular gyrus	39	L	−42	−57	51	82	−3.351
Frontal pole	9	L	−23	41	39	132	−3.606
Lateral occipital cortex	7	R	23	−59	69	80	−3.304
Lateral occipital cortex	19	L	−50	−70	−15	69	−3.267
Lingual gyrus	18	L	−14	−69	−9	82	−3.424
CD-A > HCs							
Parahippocampal gyrus	28	L	−17	−5	−32	166	4.339
Parahippocampal gyrus	35	R	18	−5	−30	96	3.499
CD-R < HCs							
Frontal orbital cortex	38	L	−45	30	−15	708	−5.166
Frontal pole	47	R	41	35	−17	588	−3.497
Temporal pole	21	R	56	12	−29	548	−4.820
CD-R > HCs							
Precentral gyrus	6	R	21	−15	63	651	3.737
Precuneous cortex	7	L	−5	−69	50	324	4.276
Subcallosal cortex	11	L	−9	18	−21	147	3.364
CD-A < CD-R							
Frontal pole	45	R	53	35	11	2034	−4.519
Postcentral gyrus	5	R	5	−36	63	331	−4.441
Frontal pole	9	L	−9	51	38	1260	−4.664
Inferior temporal gyrus	20	R	44	−5	−38	375	−4.900
Lateral occipital cortex	7	R	29	−71	47	1466	−4.401
Central opercular cortex	38	L	−39	−11	14	633	−4.232
Cingulate gyrus	24	L	−3	38	20	465	−4.324
Middle frontal gyrus	9	L	−42	24	49	474	−3.696
Superior parietal lobule	40	L	−42	−51	51	227	−3.536
Lateral occipital cortex	19	L	−30	−84	20	411	−3.944
Precuneous cortex	18	L	−8	−78	36	187	−3.911
Lingual gyrus	18	L	−15	−69	−9	146	−3.785
Supramarginal gyrus	40	L	−56	−41	35	186	−3.425
Supramarginal gyrus	42	R	57	−39	14	188	−3.505
Inferior temporal gyrus	20	R	51	−47	−14	74	−3.839

Note: The results employed gender, age, and intracranial volume as covariates. The statistical threshold was set at voxel p < 0.001, cluster p < 0.01, and two-tailed (GRF correction). CD-A refers to Crohn's disease patients in activity. CD-A refers to patients with Crohn's disease in activity. CD-R refers to patients with Crohn's disease in remission. HCs refer to Healthy controls. GMV, gray matter volume; Hem, hemisphere; BA, Brodmann area; L, left; R, right.

### 3.4 Correlation of clinical characteristics with GMV of the regional brain

**Fig. 4:**
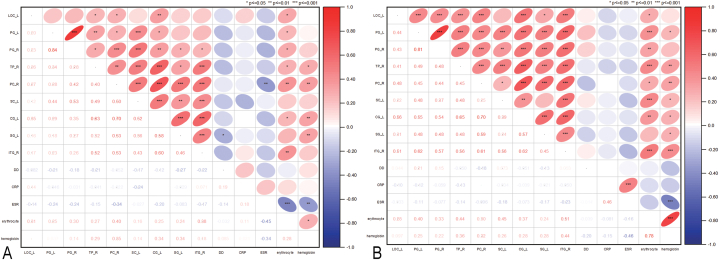
Correlation of clinical features with gray matter volume of regional brain in patients with Crohn's disease. Note: (A) The GMV in the left lateral occipital cortex, parahippocampal gyrus, right temporal pole, left precuneous cortex, left cingulate gyrus, and right inferior temporal gyrus was positive correlation with erythrocyte in the patients with Crohn's disease in activity (CD-A). The GMV in the right temporal pole, left precuneous cortex, left cingulate gyrus, and left supramarginal gyrus were positively correlated with hemoglobin in the CD-A group. (B) The GMV in the left lateral occipital cortex, parahippocampal gyrus, right temporal pole, left precuneous cortex, left subcallosal cortex, left cingulate gyrus, and right inferior temporal gyrus had a positive correlation with erythrocyte in the patients with Crohn's disease in remission (CD-R). The GMV in the left parahippocampal gyrus, right temporal pole, left precuneous cortex, left subcallosal cortex, left cingulate gyrus, left supramarginal gyrus, and right inferior temporal gyrus had a positive correlation with hemoglobin in the CD-R group.

The GMV in the left lateral occipital cortex, parahippocampal gyrus, right temporal pole, left precuneous cortex, left cingulate gyrus, and right inferior temporal gyrus was positively correlated with erythrocyte in the CD-A and CD-R group. The GMV in the right temporal pole, left precuneous cortex, left cingulate gyrus, and left supramarginal gyrus were positively correlated with hemoglobin in the CD-A and CD-R groups (Fig. 4).

### 3.5 Correlation of psychological assessment scores with GMV of the regional brain

**Fig. 5:**
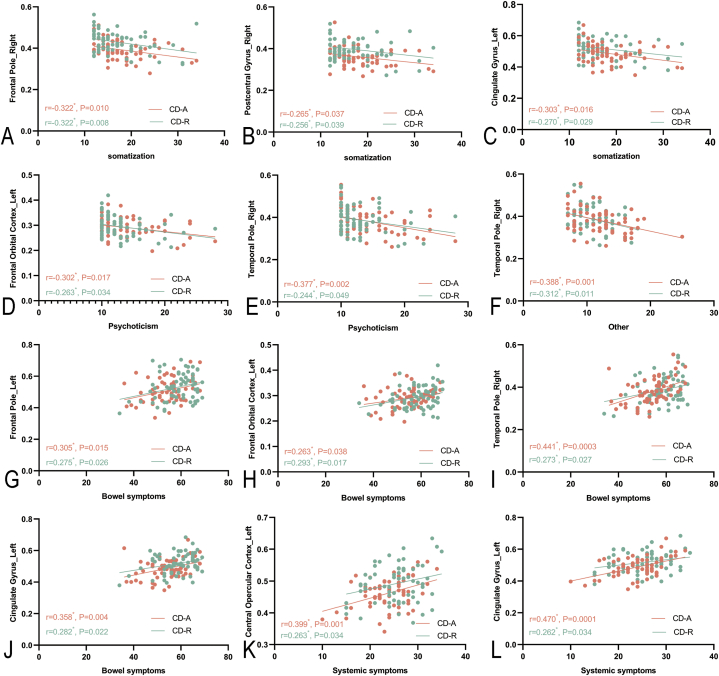
Correlation of psychological assessment features with gray matter volume of the regional brain in patients with Crohn's disease. Note: (A–C) The GMV in the right frontal pole, right postcentral gyrus, and left cingulate gyrus had a negative correlation with somatization in the patients with Crohn's disease in activity (CD-A) and in the patients with Crohn's disease in remission (CD-R). (D–E) The GMV in the left frontal orbital cortex and right temporal pole had a negative correlation with psychoticism in the CD-A and CD-R. (F) The GMV in the right temporal pole had a negative correlation with other in the CD-A and CD-R. (G–J) The GMV in the left frontal pole, left frontal orbital cortex, right temporal pole, and left cingulate gyrus were positive correlations for bowel symptoms in the CD-A and CD-R. (K–L) The GMV in the left central opercular cortex and left cingulate gyrus had a positive correlation with systemic symptoms in the CD-A and CD-R.

As shown in Fig. 5, the GMV in the right frontal pole, right postcentral gyrus, and left cingulate gyrus was negatively correlated with somatization in the CD groups. The GMV in the left frontal orbital cortex and right temporal pole were negatively correlated with psychoticism in the CD groups. The GMV in the right temporal pole was negatively correlated with other in the CD groups. The GMV in the left frontal pole, left frontal orbital cortex, right temporal pole, and left cingulate gyrus were positively correlated with bowel symptoms in the CD groups. The GMV in the left central opercular cortex and left cingulate gyrus was positively correlated with systemic symptoms in the CD groups.

## 4 Discussion

In this study, we hope to study the changes of brain gray matter volume in patients with CD by whole brain analysis. We explored morphometric brain, clinical, and psychological differences between CD-A, CD-R, and HCs using VBM. First, increased GMV of CD was observed in the parahippocampal gyrus, precentral gyrus, precuneous cortex, and subcallosal cortex, decreased was located in the occipital pole, precentral gyrus, inferior temporal gyrus, middle frontal gyrus, angular gyrus, frontal pole, lateral occipital cortex, and lingual gyrus. Second, the GMV in the right temporal pole, left precuneous cortex, and left cingulate gyrus were positively correlated with erythrocyte and hemoglobin in CD groups. The GMV in the right frontal pole, right postcentral gyrus, and left cingulate gyrus were negatively correlated with somatization in CD groups. The GMV in the right temporal pole was negatively correlated with psychoticism and other in the CD groups. The GMV in the left cingulate gyrus was positively correlated with bowel symptoms and systemic symptoms in the CD groups.

Contrary to the findings of Gita Thapaliya et al. [21], we found that the CD-A group had the largest CSF, and we also found that the disease duration in CD-R was negatively correlated with the CSF volume and the TIV. The CSF is a major component of the extracellular fluid of the CNS, which plays a significant role in intracranial pressure and blood supply. It also participates in the regulation of CSF chemical environment and plays an important role in brain metabolism. The CSF occupies the ventricles and subarachnoid space, while the interstitial fluid fills the narrow extracellular space between neurons and glial cells. Interstitial fluid and cerebrospinal fluid have been shown to exchange each other in the perivascular space. Low CSF conversion impairs brain metabolism and fluid balance early and late in life [22]. As the disease progresses, CSF turnover can be altered which could be the etiology of neurocognitive disorders [23]. The increase in SCF fluid in CD patients may be due to long disease duration caused by systemic inflammation.

The altered regions of the GMV in CD-R are partially consistent with previously reported studies [21,24,25]. The current VBM meta-analysis also showed decreased GMV in the medial frontal gyrus in patients with CD compared to HC [26]. The occipital pole is located in a triangle, the main function is to process visual information and is strongly associated with the limbic system, parietal and temporal lobes. The inferior temporal gyrus is located in an area of the brain called the temporal lobe and processes visual information. Long-term memory, emotional function, and personality traits have also been linked to frontal lobe function. As part of the anterior cingulate, the anterior cingulate is thought to contribute significantly to emotional induction and cognitive functions such as social cognition, including theoretical tasks of mind and conflict monitoring. The incoming signals from the gut are transmitted to the CNS via the spinal-cortex parallel ascending pathways [15]. The insula is widely considered to be the key to balance homeostasis input and internal response to visceral stimulation.

The decrease in GMV in those regions could be a marker of abdominal sensory perception disturbance and altered emotional evaluation of these stimuli in patients with CD. The reduction of GMV in the frontal pole in CD patients was consistent with other chronic pain disorders such as irritable bowel syndrome [27], which might be related to excitotoxicity and apoptosis due to increased cytokine release, or to astrocytes and oligodendrocytes apoptosis due to excess production of inflammatory mediators [28]. Another possible cause of reduced GMV is chronic medication, particularly corticosteroids. Appenzeller et al. found that the gray matter reduction was associated with the total corticosteroid dose in patients with systemic lupus erythematosus [29,30]. Studies have reported that the tumor necrosis factor receptor is involved in regulating the morphology of the striatum, hippocampus, and other brain regions [31]. However, we excluded patients who were actively using corticosteroids in this study. Gray matter is primarily involved in processing information in the brain and plays an important role in everyday functions such as memory, mood and movement. At present, there are still few studies on the brain structure changes in patients with CD at different active stages. This study suggests that the structural changes in the above brain areas play a certain role in different active stages.

The frontal pole and frontal orbital cortex, which were located in the interface of the higher cognitive functions and emotional regulation of the human brain, which might be associated with brain dysfunctions of sensory transmission and emotional processing in CD patients [32,33]. Meanwhile, the temporal pole is considered to help with recognizing internal changes within the body and might be associated with emotional processing and memory retrieval. The cingulate gyrus plays a role in emotional processing. The correlation between the regions of GMV and somatization and psychoticism could be attributable to the alteration of the psychological assessment of the CD patients, which was similar to the results of the previous study [34,35]. Among the regions of the GMV, the subcallosal cortex stands out under its distinctive role. One study revealed that the subcallosal cortex called the limbic system, was involved in higher nervous, mental, and visceral activities [36,37]. At the same time, the function of the lateral occipital cortex was preferentially involved in the transmission and processing of negative emotions and plays a critical role in emotional processing [38], which has been noted in several anxiety disorders. The lateral occipital cortex was associated with activated self-awareness in chronic pain patients, which was involved in self-processing tasks [39].

### 4.1 Limitation and future directions

This study has several limitations. First, this is a single-center and cross-sectional study, which is only assessed at a single time point. Thus, longitudinal research should be conducted to assess the time course of brain structural changes in CD. Second, there are variations in medication use, and disease duration of the CD groups, and should be addressed in future studies. Finally, although this study is the largest study of brain microstructural alterations to date in patients with CD, the number of participants included was not large enough for the study. In the future study, we will include more CD patients to improve the reliability of our results.

## 5 Conclusions

This is the largest study to date in patients with CD of brain gray matter microstructural alterations. We show a significant increase in global CSF volume compared with HCs, and the CSF volume was negatively correlated with the disease duration. Alterations of GMV in CD-A and CD-R and associated correlation with psychological disorders may provide evidence for possible neuromechanisms of CD with psychological disorders.

## Data and code availability statement

The materials and codes used and/or analyzed during the current study are available from the corresponding author upon reasonable request after the entire data collection procedure and project are completed.

## Statistics and biometry

No complex statistical methods were necessary for this paper.

## Ethics approval and consent to participate

The study was approved by the ethical committee of Tongji Medical College, Huazhong University of Science and Technology (Protocol Number ICH S016). All samples were collected with informed consent in accordance with the Declaration of Helsinki.

## CRediT authorship contribution statement

**Mengting Huang:** Conceptualization, Data curation, Formal analysis, Investigation, Methodology, Software, Writing – original draft, Writing – review & editing. **Hui Ma:** Conceptualization, Data curation, Formal analysis, Investigation, Resources, Software, Validation, Visualization, Writing – review & editing. **Yan Zou:** Conceptualization, Data curation, Formal analysis, Software, Validation. **Wenliang Fan:** Data curation, Methodology, Software, Supervision. **Lei Tu:** Formal analysis, Funding acquisition, Investigation, Resources, Supervision, Validation. **Jie Zhao:** Formal analysis, Project administration, Resources, Software, Supervision, Validation, Visualization. **Guina Ma:** Investigation, Methodology, Supervision, Validation, Visualization, Writing – review & editing. **Nan Diao:** Investigation, Methodology, Resources, Software, Visualization, Writing – review & editing. **Xin Li:** Conceptualization, Data curation, Formal analysis, Investigation, Methodology, Resources, Software, Supervision, Validation, Visualization. **Ping Han:** Conceptualization, Data curation, Formal analysis, Funding acquisition, Methodology, Software, Supervision, Validation, Visualization, Project administration, Resources, Writing – review & editing. **Liangru Zhu:** Conceptualization, Data curation, Formal analysis, Funding acquisition, Investigation, Methodology, Project administration, Resources, Software, Supervision, Validation, Visualization, Writing – review & editing. **Heshui Shi:** Conceptualization, Data curation, Formal analysis, Funding acquisition, Investigation, Methodology, Project administration, Resources, Software, Supervision, Validation, Visualization, Writing – review & editing.

## Declaration of competing interest

The authors declare that they have no known competing financial interests or personal relationships that could have appeared to influence the work reported in this paper.
