# Supplementary material for: Structural alterations of brain in different disease states of Crohn's disease: Results of a cross-sectional study in a Chinese hospital
Source: Heliyon. 2024 Mar 7;10(6):e27446. doi: 10.1016/j.heliyon.2024.e27446 (PMC10951496; doi:10.1016/j.heliyon.2024.e27446)
Supplement: Multimedia component 1 [file mmc1.docx]

**Supplementary Material Table 1**

Questionnaire assessment data of all participants.

| Characteristics | CD-A (n=62) | CD-R (n=65) | HCs (n=92) | P |
| --- | --- | --- | --- | --- |
| IBDQ | - | - |  | - |
| Bowel symptoms | 54.34±7.45^***^ | 58.45±7.49^&&&^ | 64.22±2.31^###^ | <0.0001 |
| Systemic symptoms | 23.94±4.67 | 25.48±4.91^&&&^ | 29.00±1.42^###^ | <0.0001 |
| Emotional function | 58.94±9.59^***^ | 64.06±9.17^&&&^ | 77.00±3.91^###^ | <0.0001 |
| Social impairment | 23.71±6.67^**^ | 26.80±5.76^&&&^ | 35.42±4.61^###^ | <0.0001 |
| SSRS | - | - |  | - |
| Objective support | 8.82±2.71 | 9.43±3.00^&&&^ | 44.02±1.35^###^ | 0.234 |
| Subjective Support | 15.82±5.76 | 17.37±5.57^&&&^ | 11.31±1.56^###^ | 0.126 |
| Availability | 6.95±1.71 | 7.24±1.81^&&&^ | 10.13±0.96^###^ | 0.348 |
| SCL-90 | - | - |  | - |
| Somatization | 18.26±5.03^*^ | 17.17±5.35 | 14.29±4.44^###^ | <0.0001 |
| Obsessive-Compulsive | 17.77±5.26 | 16.37±4.86^&&^ | 13.99±4.18^###^ | <0.0001 |
| Interpersonal sensitivity | 14.92±5.65^**^ | 12.46±4.03^&^ | 10.68±1.97^###^ | <0.0001 |
| Depression | 23.79±8.67^***^ | 19.69±5.98^&&&^ | 15.84±3.76^###^ | <0.0001 |
| Anxiety | 15.05±5.07 | 13.85±3.91^&&&^ | 11.58±2.30^###^ | <0.0001 |
| Hostility | 9.95±3.61 | 9.09±3.36^&&&^ | 6.89±1.26^###^ | <0.0001 |
| Phobic anxiety | 9.19±3.02 | 8.46±2.07 | 7.67±1.06^###^ | <0.0001 |
| Bigoted | 8.79±2.90^***^ | 7.38±2.25^&^ | 6.59±0.97^###^ | <0.0001 |
| Psychoticism | 14.32±4.36 | 13.08±3.68^&&&^ | 11.11±1.56^###^ | <0.0001 |
| Other | 12.03±3.68 | 10.85±2.75^&&&^ | 8.64±2.35^###^ | <0.0001 |

Note: CD-A refers to patients with Crohn’s disease in activity. CD-R refers to patients with Crohn’s disease in remission. HCs refer to Healthy controls. IBDQ, Inflammatory bowel disease questionnaire; SCL-90, Symptom checklist-90; SSRS, Social Support Rating Scale;

P value for One-way ANOVA.

* Indicates a significant difference between CD-A and CD-R (P < 0.05, P<0.01, P<0.001).

# Indicates a significant difference between CD-A and HCs (P < 0.05, P<0.01, P<0.001).

& Indicates a significant difference between CD-R and HCs (P < 0.05, P<0.01, P<0.001).

**Supplementary Material 2: Inflammatory Bowel Disease Questionnaire (IBDQ)**

1.How frequent have your bowel movements been during the last two weeks?

a) bowel movements as or more frequent than they have ever been; b) extremely frequent; c) very frequent; d) moderate increase in frequency of bowel movements; e) some increase in frequency of bowel movements; f) slight increase in frequency of bowel movements; g) normal.

The working structure of the other questions is identical, and appropriate seven-point scales are offered for each question. 1- all the time; 2- most of the time; 3- lots of time; 4-some time; 5- a fraction of the time; 6- very little time; 7-None.

The content of the remaining 31 questions is as follows:

2. In the past 2 weeks, how often have you been affected by fatigue, weakness, or burnout?

3. In the past 2 weeks, how often have you felt frustrated, impatient, or irritable?

4. In the past 2 weeks, how many times have you been unable to attend school or work due to intestinal problems?

5. In the past 2 weeks, how many times have you had loose stools?

6. How energetic have you been over the past two weeks?

7. In the past 2 weeks, how often have you spent worrying that your bowel problem might require surgery?

8. In the past 2 weeks, how often have you had to postpone or cancel social events because of intestinal problems?

9. In the past 2 weeks, how often have you had trouble with abdominal cramps?

10. In the past 2 weeks, how often have you felt unwell?

11. In the past 2 weeks, how often have you spent worrying about finding a toilet?

12. In the past 2 weeks, how difficult has your bowel problem been to participate in leisure or sports?

13. In the past 2 weeks, how often have you been bothered by abdominal pain?

14. In the past 2 weeks, how often have you been bothered by not being able to sleep at night or waking up during the night?

15. In the past 2 weeks, how often have you felt depressed or depressed?

16. In the past 2 weeks, how often have you had to avoid attending events where there was no washroom close at hand?

17. In the last 2 weeks, how much of a problem have you had with passing large amounts of gas?

18. In the past 2 weeks, how much of a problem has it been for you to maintain or reach your ideal weight?

19. Many patients with intestinal disease often have worries and anxieties related to their illness. Overall, in the past 2 weeks, how often have you had felt worried or anxious?

20. In the past 2 weeks, how often have you had to worry about bloating?

21. In the past 2 weeks, how often have you felt relaxed and stress-free?

22. In the past 2 weeks, how often have you had problems with rectal bleeding with your bowel movements?

23. In the past 2 weeks, how often have you been embarrassed by your bowel problems?

24. In the past 2 weeks, how often have you been troubled by a feeling of having to go to the bathroom even though your bowels are empty?

25. In the past 2 weeks, how often have you felt tearful or upset?

26. In the past 2 weeks, how often have you been troubled by accidental soiling of your underpants?

27. In the past 2 weeks, how often have you felt angry as a result of your bowel problem?

28. In the past 2 weeks, what extent has your bowel problem limited sexual activity?

29. In the past 2 weeks, how often have you been troubled by feeling sick to your stomach?

30. In the past 2 weeks, how often have you felt irritable?

31. In the past 2 weeks, how often have you felt lack of understanding from others?

32. In the past 2 weeks, how satisfied, happy, or pleased have you been with your personal life?

**Supplementary Material 3: Social support rating scale (SSRS)**

The following questions are intended to reflect the support you have received in the community. Please answer them according to the specific requirements of each question and according to your actual situation.

1. How many close friends do you have that you can rely on for support or help? a) Noe; b) 1-2; c) 3-5; d) More than 5.

2. For nearly a year you? a) Away from family; b) The residence changes frequently, and most of the time living with strangers; c) Living with classmates, colleagues, or friends; d) Living with family.

3. You and your neighbor? a) Never care about each other, just nodding acquaintances; b) May be a little concerned when encountering difficulties; c) Some of your neighbors are concerned about you; d) Most of the neighbors care about you.

4. You and your colleagues? a) Never care about each other, just nodding acquaintances; b) May be a little concerned when encountering difficulties; c) Some of your colleagues are concerned about you; d) Most of the colleagues care about you.

5.1 Support and care from a couple/lover? a) None; b) Very few; c) In general; d) Full support;

5.2 Support and care from parents? a) None; b) Very few; c) In general; d) Full support;

5.3 Support and care from children? a) None; b) Very few; c) In general; d) Full support;

5.4 Support and care from brothers and sisters? a) None; b) Very few; c) In general; d) Full support;

5.5 Support and care from other family members? a) None; b) Very few; c) In general; d) Full support;

6. What sources of financial or problem-solving help have you received in the past? (1) No source; (2) The following sources: a) Spouse; b) Other family; c) Friend; d) Relative; e) Colleague; f) Work unit;

7. What sources of comfort and concern have you received in emergency situations? (1) No source; (2) The following sources: a) Spouse; b) Other family; c) Friend; d) Relative; e) Colleague; f) Work unit;

8. What is your way of talking when you are troubled? a) Don't want anyone to talk to; b) Only confide in 1-2 people who are very close to you; c) If a friend asks, you will speak up; d) Open up about your troubles to gain support and understanding;

9. What is your recourse when you are in trouble? a) By myself; b) Rarely asks for other help; c) Ask others for help sometimes; d) Always ask for help from family, friends and relatives, and organizations.

10. How often do you participate in activities organized by groups (such as party organizations, religious organizations, trade unions, student unions)? a) Never; b) Occasionally; c) Regularly; d) Actively.

**Supplementary Material 4: Symptom checklist-90 (SCL-90)**

Each item is scored on a scale from 0 to 4 based on how much an individual was bothered by each item in the last week: 0 = Not at all; 1 = A little bit; 2 = Moderately; 3 = Quite a bit; 4 = Extremely.

The content of the remaining 90 questions is as follows:

1. Headaches; 2. Nervousness or shakiness inside; 3. Unwanted thoughts or ideas that won’t leave your head; 4. Faintness or dizziness; 5. Loss of sexual interest or pleasure; 6. Feeling critical of others; 7. The idea that someone else can control your thoughts; 8. Feeling others are to blame for most of your troubles; 9. Trouble remembering things; 10. Worried about sloppiness or carelessness; 11. Feeling easily annoyed or irritated; 12. Pains in heart or chest; 13. Feeling afraid in open spaces or on the street;

14 Feeling low in energy or slowed down; 15. Thoughts of ending life; 16. Hearing voices that other people do not hear; 17. Trembling; 18. Feeling that most people cannot be trusted; 19. Poor appetite; 20. Crying easily; 21. Feeling shy or uneasy with the opposit sex; 22. Feeling of being trapped or caught;

23. Suddenly scared for no reason; 24. Temper outbursts that you could not control; 25. Feeling afraid to go out of your house alone; 26. Blaming yourself for things; 27. Pains in lower back; 28. Feeling blocked in getting things done; 29. Feeling lonely; 30. Feeling blue; 31. Worrying too much about things;

32. Feeling no interest in things; 33. Feeling fearful; 34. Your feelings being easily hurt; 35. Other people being aware of your private thoughts; 36. Feeling others do not understand you or are unsympathetic; 37. Feeling that people are unfriendly; 38. Having to do things very slowly; 39. Heart pounding or racing; 40. Nausea or upset stomach; 41. Feeling inferior to others; 42. Soreness of your muscles; 43. Feeling that you are watched or talked about by others; 44. Trouble falling asleep; 45. Having to check and double check what you do; 46. Difficulty making decisions; 47. Feeling afraid to travel on buses, subways or trains; 48. Trouble getting your breath; 49. Hot or cold spells; 50. Having to avoid certain things, places or activities; 51. Your mind going blank; 52. Numbness or tingling in parts of your body; 53. A lump in your throat; 54. Feeling hopeless about the future; 55. Trouble concentrating; 56. Feeling weak in parts of your body; 57. Feeling tense or keyed up; 58. Heavy feelings in your arms or legs; 59. Thoughts of death or dying; 60. Overeating; 61. Feeling uneasy when people are watching or talking about you; 62. Having thoughts that are not your own; 63. Having urges to beat, injure or harm someone; 64. Awakening in the early morning; 65. Having to repeat the same actions such as touching, counting, washing; 66.Sleep that is restless or disturbed; 67. Having urges to break or smash things; 68. Having ideas or beliefs that others do not share; 69. Feeling very self-conscious with others; 70. Feeling uneasy in crowds such as shopping or at a movie; 71. Feeling everything is an effort;

72. Spells of terror or panic; 73. Feeling uncomfortable about eating or drinking in public; 74. Getting into frequent arguments; 75. Feeling nervous when you are left alone; 76. Others not giving you proper credit for your achievments; 77. Feeling lonely even when you are with people; 78. Feeling so restless you couldn’t sit still; 79. Feeling of worthlessness; 80. Feeling that familiar things are strange or unreal;

81. Shouting or throwing things; 82. Feeling afraid you will faint in public; 83. Feeling that people will take advantage of you if you let them; 84. Having thoughts about sex that bother you a lot; 85. The idea that you should be punished for your sins; 86. Feeling pushed to get things done; 87. The idea that something serious is wrong with your body; 88. Never feeling close to another; 89. Feelings of guilt; 90. The idea that something is wrong with your mind.
